# Supplementary material for: Lipase-mediated detoxification of host-derived antimicrobial fatty acids by Staphylococcus aureus
Source: Commun Biol. 2024 May 15;7:572. doi: 10.1038/s42003-024-06278-3 (PMC11096360; doi:10.1038/s42003-024-06278-3)
Supplement: Supplementary file 2 — Supplementary Information [file 42003_2024_6278_MOESM2_ESM.pdf]

Supplementary Information

Lipase-mediated detoxification of host-derived antimicrobial fatty acids by *Staphylococcus aureus*

Arnaud Kengmo Tchoupa[1,2,3], Ahmed M. A. Elsherbini[1,2,3,#], Justine Camus[1,2,3,#], Xiaoqing Fu[4], Xuanheng Hu[1,2,3], Oumayma Ghaneme[1,2,3], Lea Seibert[1,2,3], Marco Lebtig[1,2,3], Marieke A. Böcker[1,2,3], Anima Horlbeck[1,2,3], Stilianos P. Lambidis[1,2,3], Birgit Schittek[2,5], Dorothee Kretschmer[1,2,3], Michael Lämmerhofer[4], Andreas Peschel[1,2,3]

[1] Interfaculty Institute of Microbiology and Infection Medicine Tübingen, Infection Biology Section, University of Tübingen, Tübingen, Germany

[2] Cluster of Excellence EXC 2124 Controlling Microbes to Fight Infections, University of Tübingen, Tübingen, Germany

[3] German Center for Infection Research (DZIF), partner site Tübingen

[4] Institute of Pharmaceutical Sciences, University of Tübingen, Tübingen, Germany

[5] Dermatology Department, University Hospital Tübingen, Tübingen, Germany

[#] These authors contributed equally

Correspondence to

Arnaud Kengmo Tchoupa (arnaud.kengmo-tchoupa@uni-tuebingen.de)

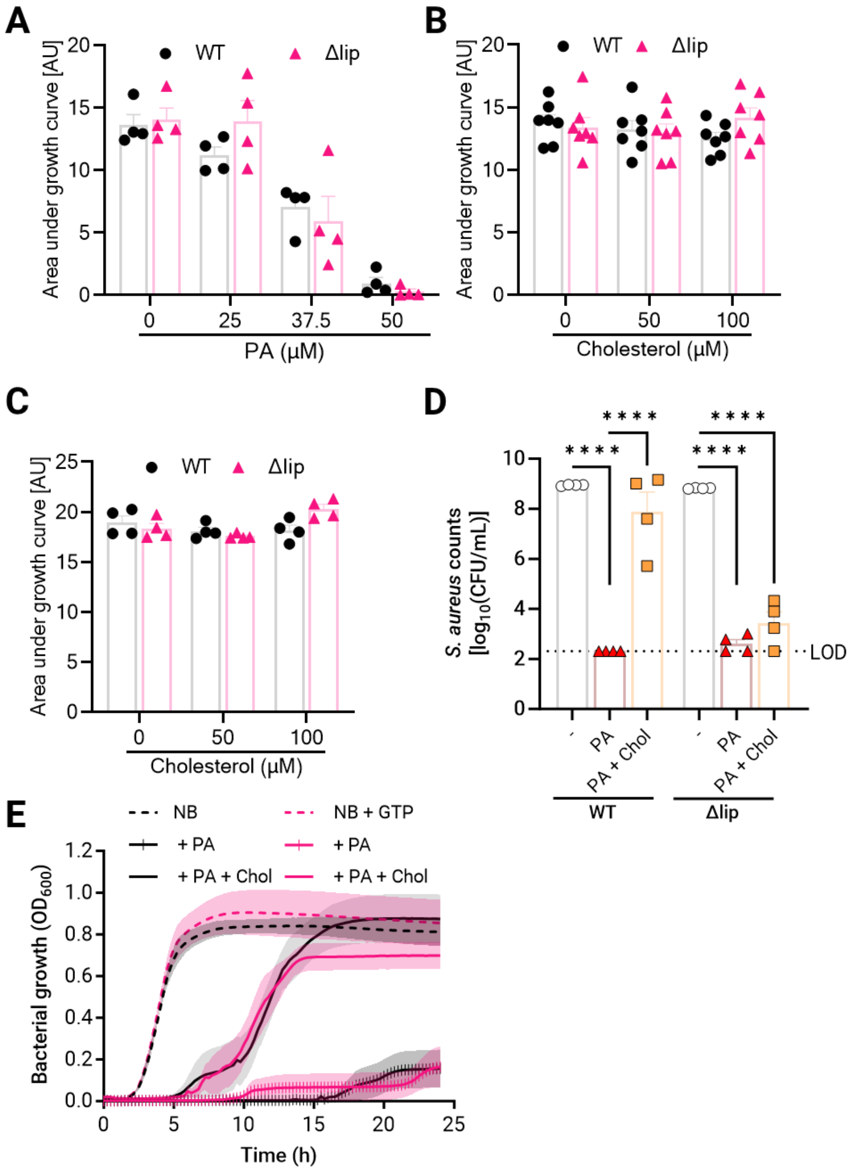

Supplementary Figure 1. Cholesterol-dependent protective roles of *S. aureus* lipases against palmitoleic acid.

A. USA300 JE2 (WT) and its Lip1- and Lip2-defective double mutant (Δlip) were grown in nutrient broth (NB) supplemented with 0 to 50 µM palmitoleic acid (PA). Computed area under growth curves was plotted. **B-C**. Area under the curves of WT and Δlip upon growth in NB (**B**) or tryptic soy broth (TSB) (**C**) supplemented with 0, 50, or 100 µM cholesterol (Chol). **D.** Viable WT and Δlip were enumerated upon growth for 24 h in chemically defined medium (CDM), or CDM supplemented with 50 µM PA or 50 µM PA + 50 µM cholesterol (Chol). **E.** WT was grown in plain nutrient broth (NB), or NB + 50 µM glycerol tripalmitoleate (GTP). NB or NB + GTP was supplemented with 50 µM palmitoleic acid (PA) or 50 µM PA and 50 µM cholesterol (Chol). Optical density at 600 nm (OD_{600}) was measured over 24 h. Statistical significance was determined by one-way analysis of variance (ANOVA) with Tukey's multiple comparisons test. ****P < 0.0001. LOD (limit of detection) = 203 CFU.

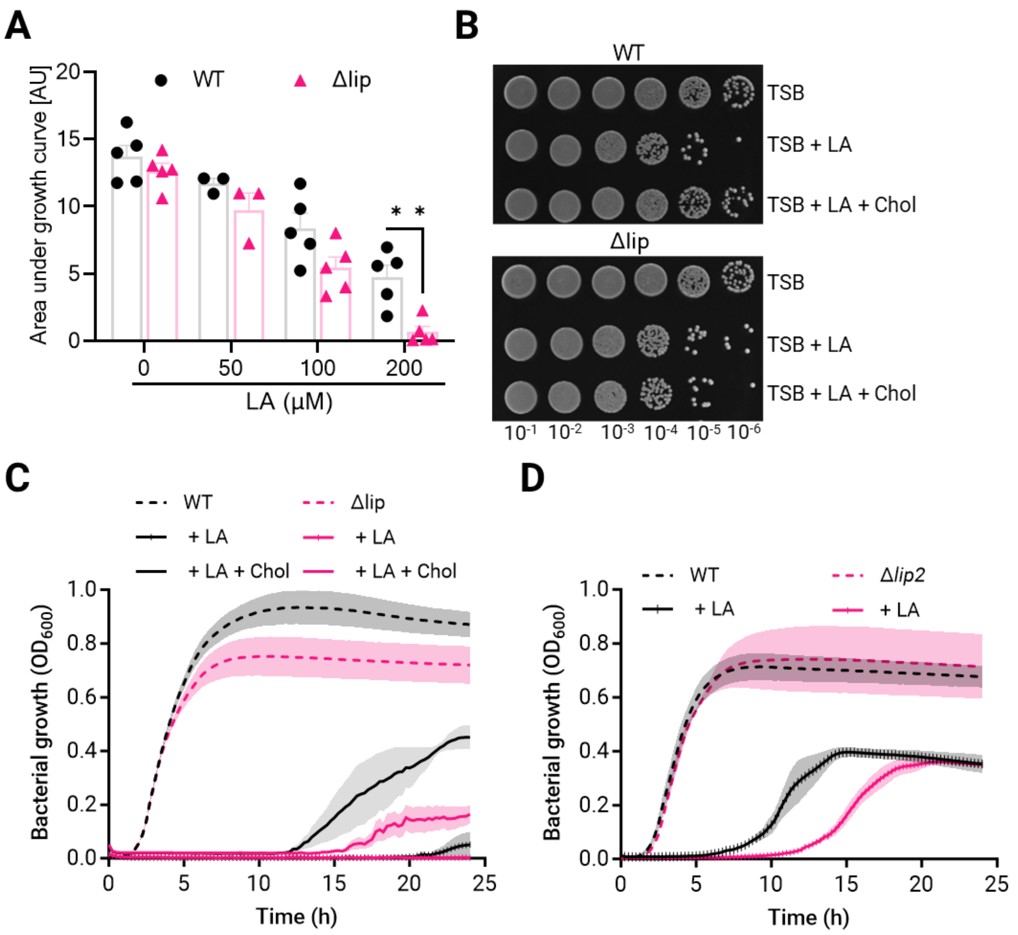

Supplementary Figure 2. Lipase deficiency increases susceptibility to linoleic acid.

A. USA300 JE2 (WT) and its Lip1- and Lip2-defective double mutant (Δlip) were grown in nutrient broth (NB) or NB plus linoleic acid (LA). Computed area under growth curves was plotted. **B**. WT and Δlip were spotted onto TSB upon growth for 24 h in TSB, or TSB supplemented with 200 µM LA or 200 µM LA and 100 µM cholesterol (Chol). **C.** WT and Δlip were grown to the exponential phase prior to growth assays in NB or NB supplemented with 100 µM LA or 100 µM LA and 100 µM cholesterol **D**. The growth of the Lip2 mutant (Δ*lip2*) or isogenic wild-type USA300 (WT) was monitored over 24 h by OD_{600} readings in NB or NB supplemented with 200 µM LA. Data shown are means ± SEM for at least three biological replicates. Statistical significance was evaluated by two-way ANOVA with Šídák's multiple comparisons test. ***P* = 0.0049.

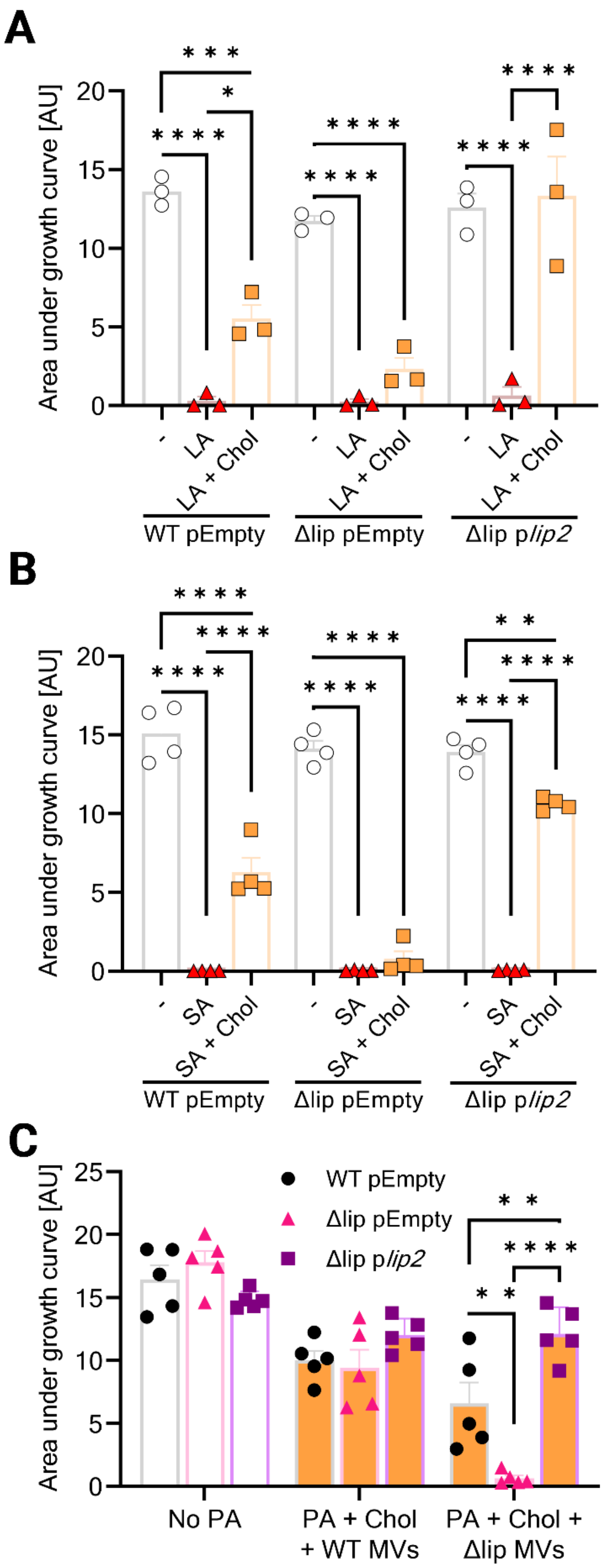

Supplementary Figure 3. The lipase Lip2 is required for cholesterol-mediated protection against AFAs.

A. Wild-type USA300 JE2 (WT) and its isogenic Δlip mutant bearing pEmpty, and Δlip complemented with p*lip2* were grown in plain NB, or NB supplemented with 150 µM LA or 150 µM LA and 75 µM Chol. Growth was computed as area under the curves. **B-C**. Area under the curves of the strains described in **A** upon growth in NB, or NB supplemented with 50 µM sapienic acid (SA), 50 µM SA + 50 µM Chol (**B**), or 50 µM palmitoleic acid (PA) + 50 µM Chol and in the presence of membrane vesicles (MVs) from WT or Δlip (**C**). Data shown are means + SEM for three (**A**), four (**B**) or five (**C**) biological replicates. Statistical significance was evaluated by one- (**A**, **B**) or two-way (**C**) ANOVA with Tukey's multiple comparisons test. **P* < 0.05, ***P* < 0.01, ****P* < 0.0006, *****P* < 0.0001.

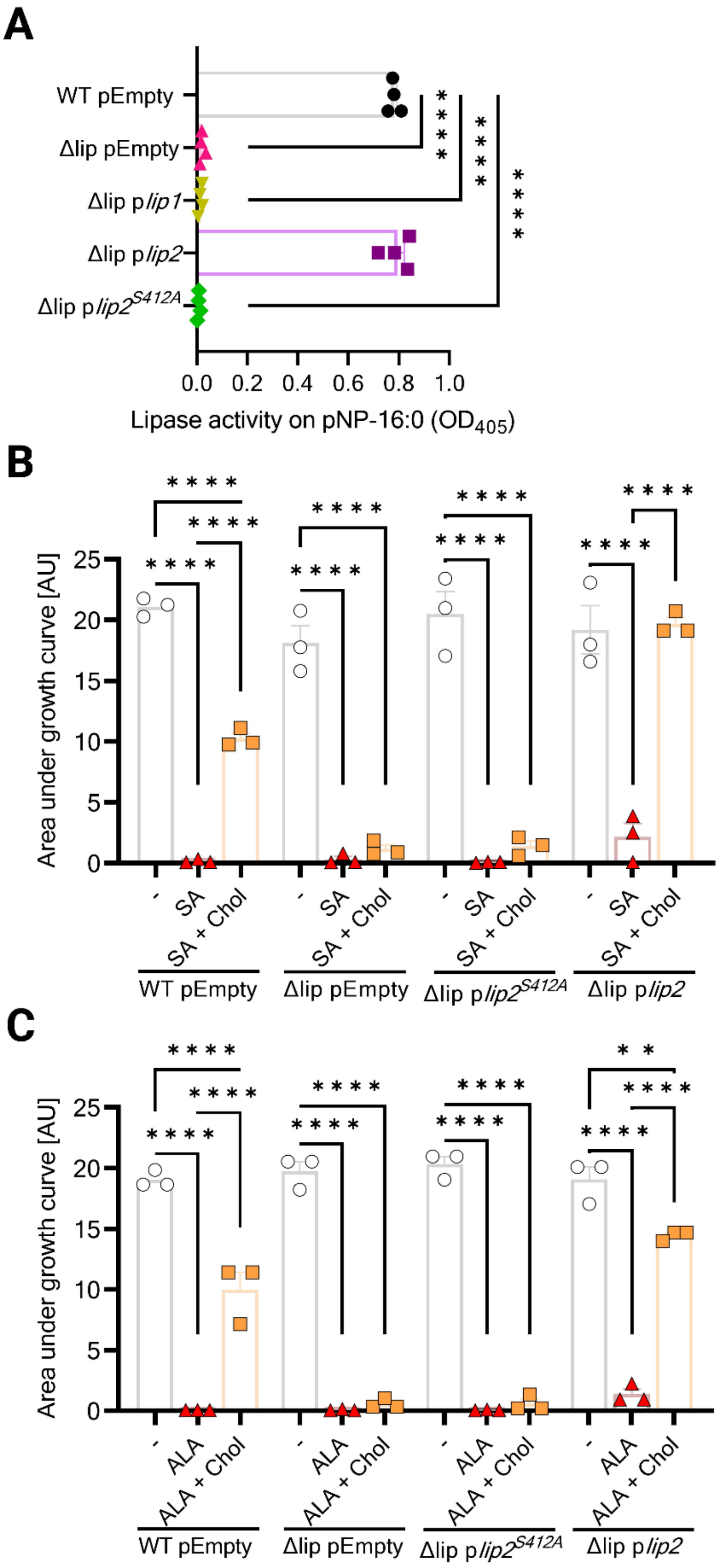

Supplementary Figure 4. Inactivation of Lip2 abrogates cholesterol protection against AFAs.

A. The *S. aureus*-conditioned media from the indicated strain (WT pEmpty, Δlip pEmpty, Δlip p*lip1*, Δlip p*lip2*, or Δlip p*lip2*^{S412A}) were incubated with *para*-nitrophenyl palmitate (pNP-16:0). The release of *para*-nitrophenol, indicative of lipase activity, was quantified by measuring OD_{405}. **B-C**. pEmpty-bearing wild-type USA300 JE2 and its isogenic Δlip mutant, and Δlip complemented with either p*lip2*^{S412A} or p*lip2* were grown in BM, or BM supplemented with AFA or AFA + Chol. AFA was sapienic acid (SA) or α-linolenic acid (ALA) in (B) and (C), respectively. Area under growth curves was plotted. Shown are means + SEM for three or four biological replicates. One-way ANOVA with Dunnett's test relative to WT pEmpty (**A**) or Tukey's multiple comparisons test (**B**, **C**) was used to calculate statistical significance. $^{**}P = 0.0011$, $^{****}P < 0.0001$.

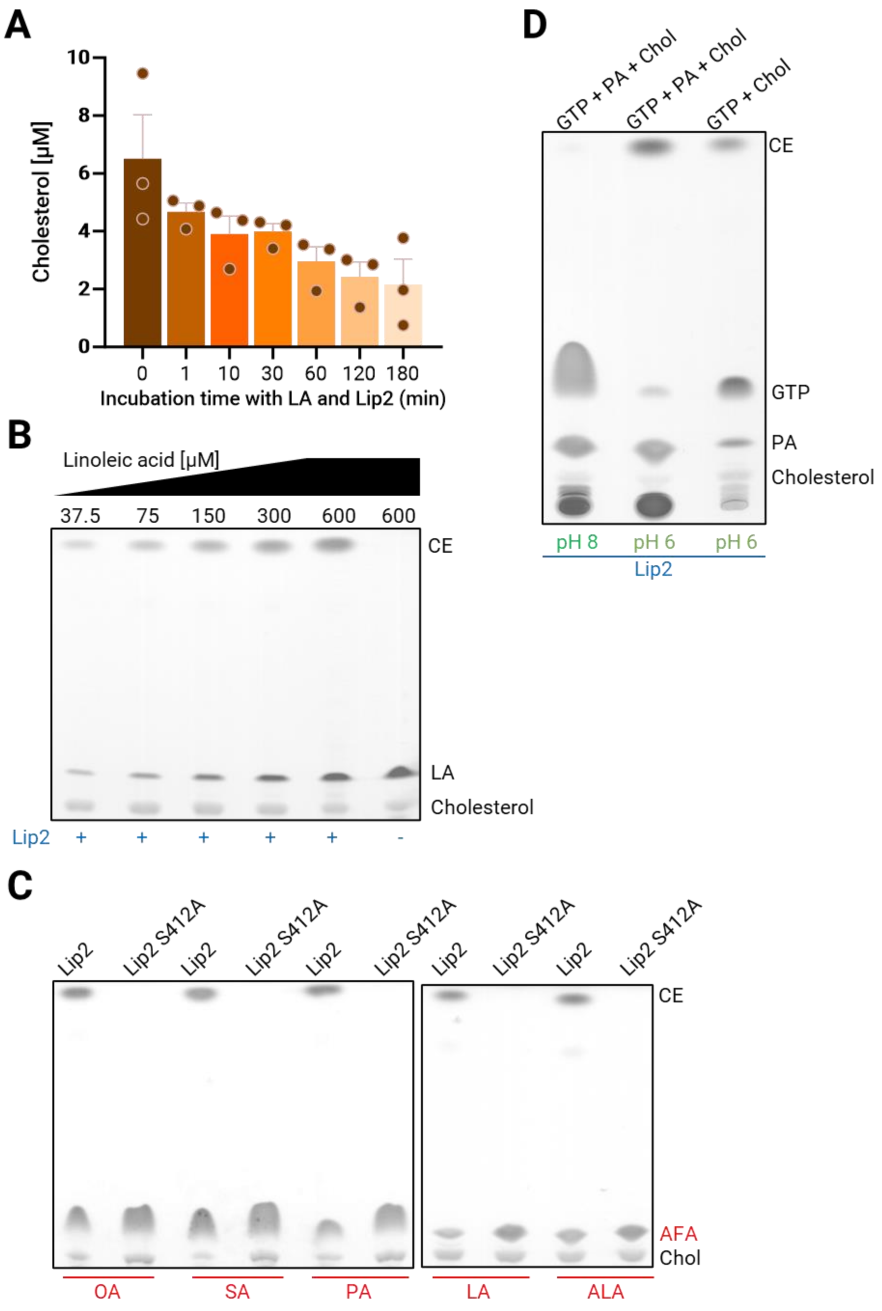

Supplementary Figure 5. Recombinant Lip2 esterifies AFAs with cholesterol.

A. Free cholesterol was quantified in mixtures of 30 µM cholesterol (Chol) with 30 µM linoleic acid (LA) before (t = 0) or following the addition of recombinant *Staphylococcus aureus* lipase 2 (Lip2; 1.4 ng/mL) to the sample and incubation for the indicated duration. **B**. Thin layer chromatography (TLC) of lipid extracts upon incubation of increasing concentrations of LA with Chol in the presence or absence of Lip2. **C**. TLC of lipids extracted after incubation of OA (oleic acid), SA (sapienic acid), PA (palmitoleic acid), LA, or ALA (α-linolenic acid) with Chol in the presence of Lip2 or catalytically dead Lip2 S412A. Cholesteryl esters (CE) were detected for all AFAs tested. **D.** TLC of lipid extracts following incubation of Lip2, glycerol tripalmitoleate (GTP), and cholesterol in the presence or absence of PA at the indicated pH.

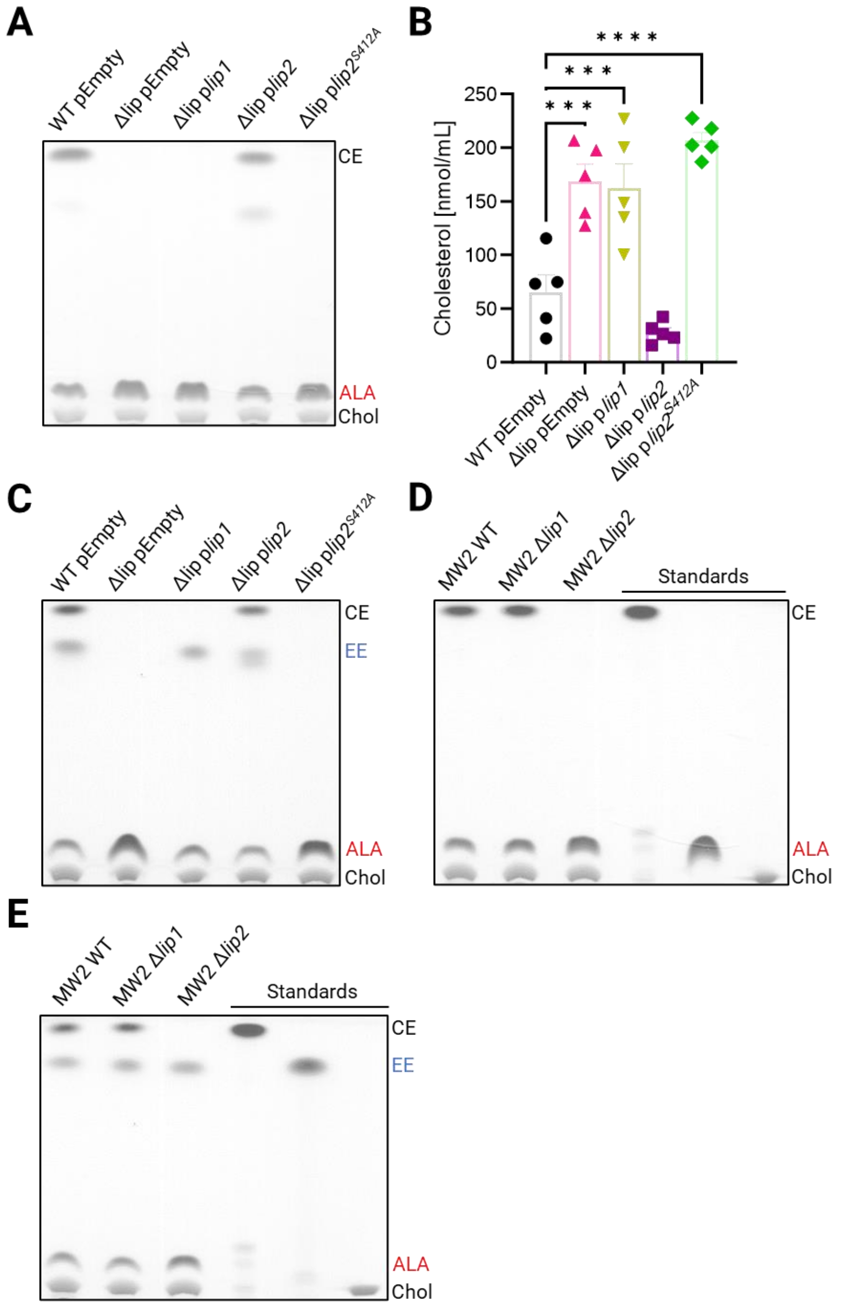

Supplementary Figure 6. Esterification activities of lipases from *S. aureus*-conditioned media.

A. Thin layer chromatography (TLC) lipid analysis of USA300 *S. aureus*-conditioned media from the indicated strain (WT pEmpty, Δlip pEmpty, Δlip p*lip1*, Δlip p*lip2*, or Δlip p*lip2*^{S412A}) incubated with cholesterol (Chol) and α-linolenic acid (ALA). **B**. UHPLC-MS/MS lipid analysis to measure cholesterol upon incubation of *S. aureus*-conditioned media from strains described in (**A**) with Chol and linoleic acid. **C**. TLC of lipids extracted after incubation of *S. aureus*-conditioned media from strains listed in (**A)** with Chol, ethanol, and ALA. Ethyl esters (EE) and/or cholesteryl esters (CE) were detected. **D-E**. TLC lipid analysis of *S. aureus*-conditioned media from wild-type USA400 MW2 (MW2 WT), or its lipase-deficient mutants (MW2 Δ*lip1* and MW2 Δ*lip2*) incubated with Chol and ALA in the absence (**D**) or presence of ethanol (**E**). Four lipid standards (cholesterol, ALA, ethyl ALA, and cholesteryl ALA) are shown. Bar graphs (**B**) are means + SEM for five biological replicates. Statistical significance by one-way ANOVA with Dunnett's test relative to WT pEmpty. ***P < 0.001, ****P < 0.0001.

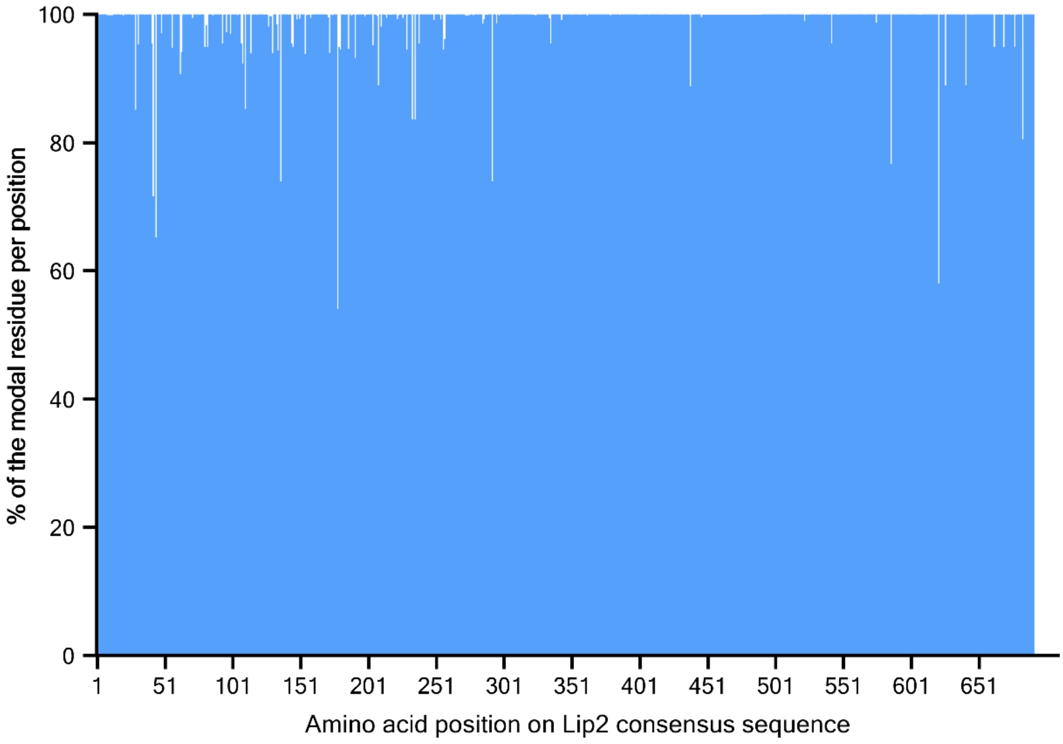

Supplementary Figure 7. Lip2 is conserved in *S. aureus*.

Lip2 is generally synthesized as a 690 or 691 amino acid polypeptide. A consensus Lip2 sequence was generated upon alignment of over 3000 Lip2 sequences from our database to USA300 Lip2 as reference. The percentage of the modal residue at each amino acid position is shown.

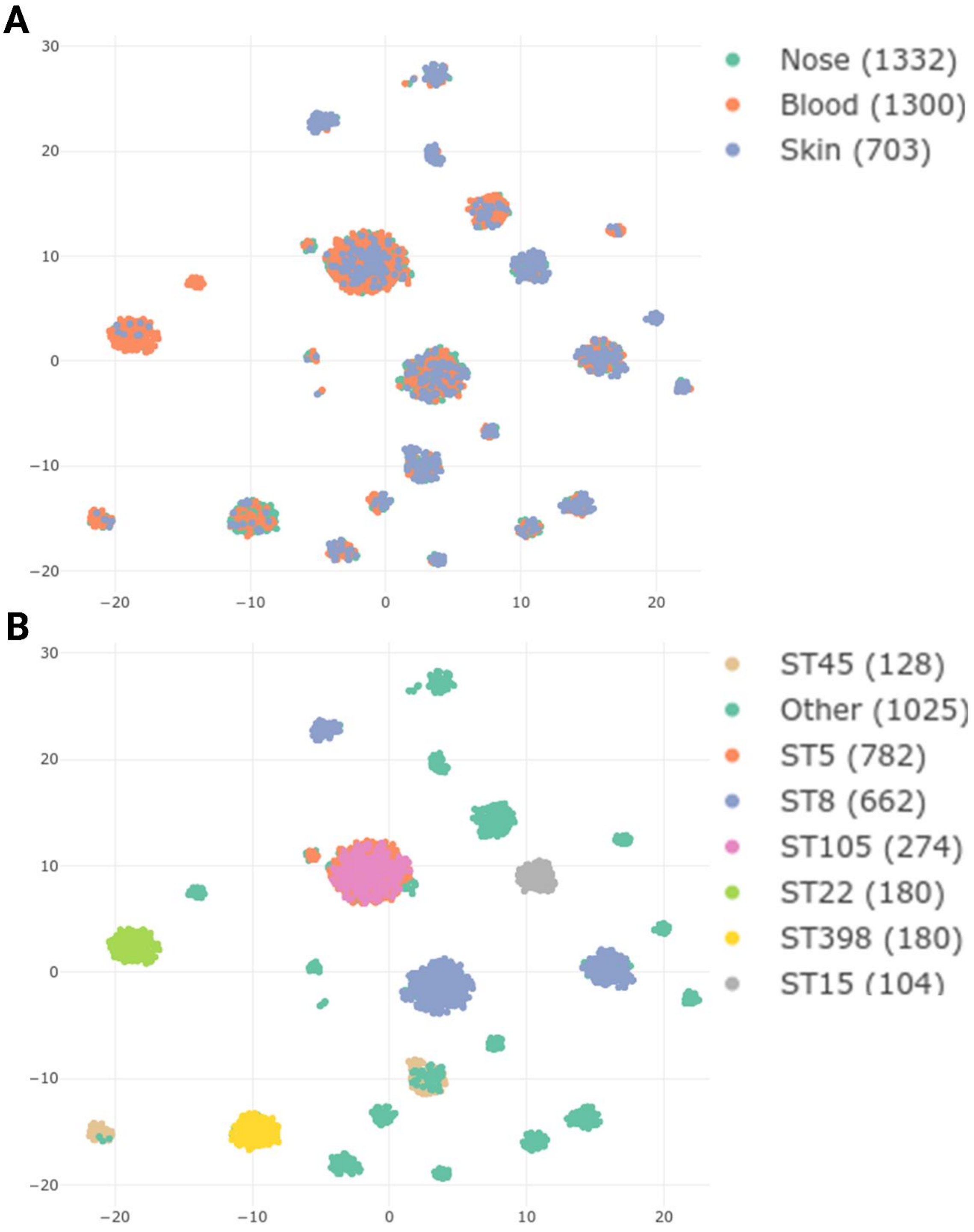

Supplementary Figure 8. The sequence type dictates Lip2 diversity.

The multiple sequence alignment of over 3000 Lip2 sequences is represented as three-dimensional space generated using dimensionality reduction. Lip2 sequence of each *S. aureus* strain is represented as a dot whose color depends either on the isolation site (**A**) or the sequence type (ST) (**B**) of the bacterium.

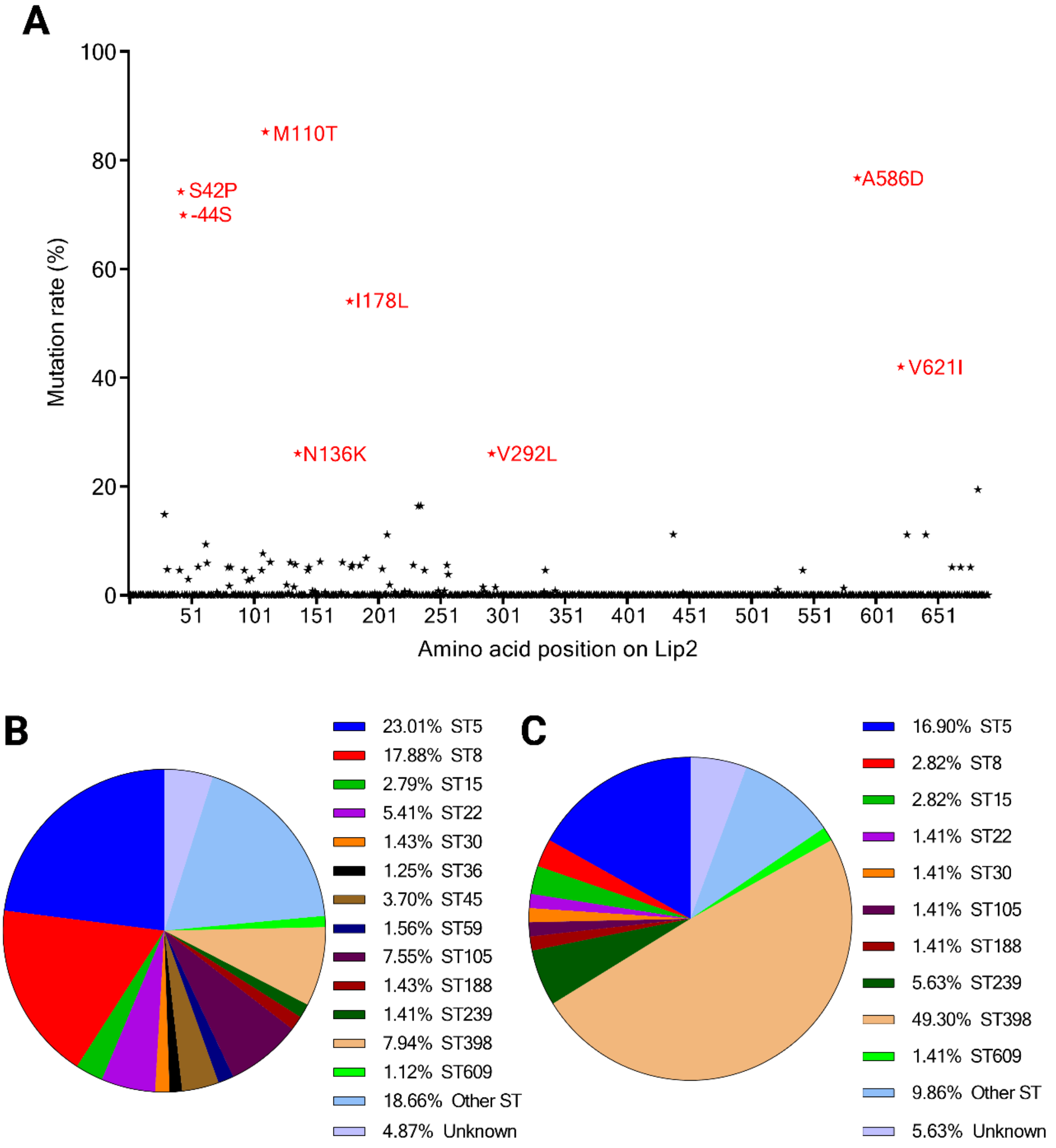

Supplementary Figure 9. Lip2 displays mutation hotspots and is disproportionally disrupted in ST398 strains.

A. Lip2 is usually a 690 or 691 amino acid protein. For the > 3000 Lip2 sequences from our database, the mutation rate at each amino acid position, relative to USA300, was determined. The insertion of serine (S) between positions 43 and 44 in ~ 70% of our strains is denoted as "-44S" and highlighted in red as well as all mutations that occurred in at least a quarter of our database. **B-C**. Sequence types (ST) of all *S. aureus* isolates in our database (**B**) or isolates with prophage-disrupted Lip2 (**C**).

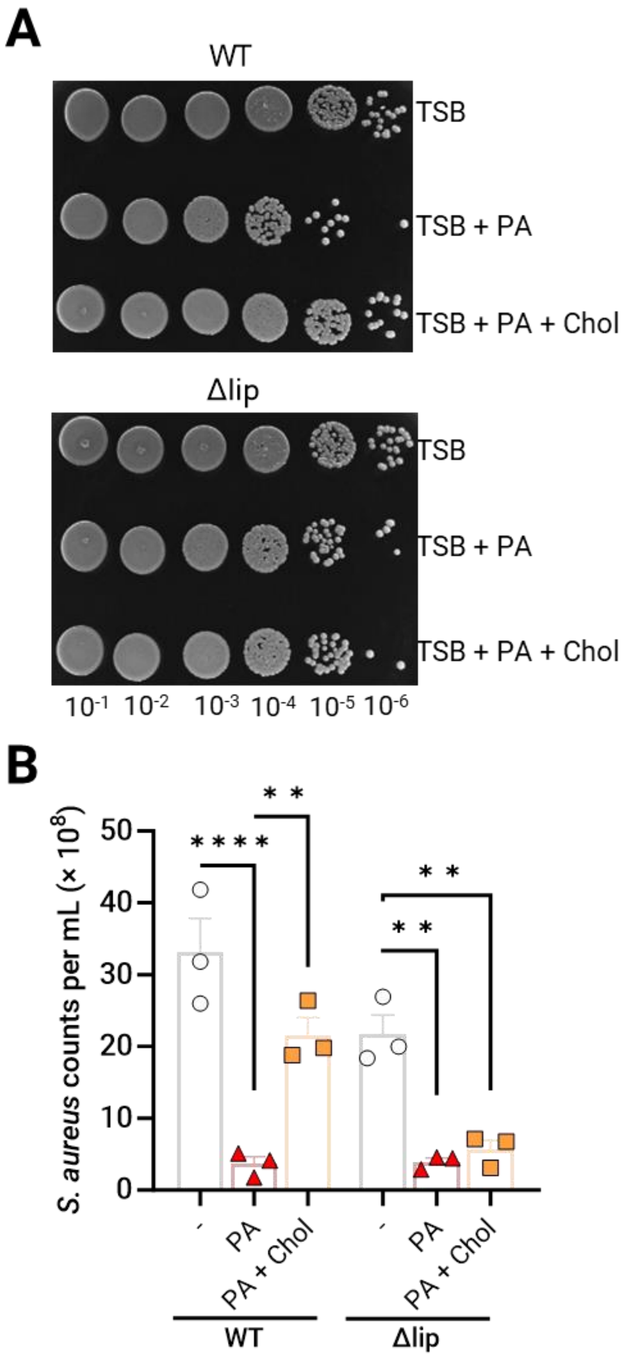

Supplementary Figure S10. Washed *S. aureus* is still protected from AFAs by cholesterol.

A-B. USA300 JE2 (WT) and its Lip1- and Lip2-defective double mutant (Δlip) were washed in PBS prior growth for 24 h in tryptic soy broth (TSB) or TSB supplemented with 150 µM palmitoleic acid (PA) or 150 µM PA and 150 µM cholesterol (Chol). Cultures were then serially diluted and spotted onto TSB plates. Representative pictures of the spots (**A**) or CFU data are shown (**B**). Bar graphs (**B**) are means + SEM for three biological replicates. Statistical significance was evaluated by one-way ANOVA with Tukey's multiple comparisons test. $^{**}P < 0.001$; $^{****}P < 0.0001$.

Supplementary Table 1: Mutations co-occurring in Lip2

Mutations	Strain number
S42P,-44S,M110T,I178L,A586D,V621I	1174
M110T	199
V29I,S42P,-44S,I63V,I108S,M110T,Q130P,N136K,G154E,I172V,P191L,T208S,T233N,R235H,V292I, K438Q,A586D,D626N,I641L,S683G,	190
V29I,S42P,-44S,A62T,M110T,T134I,N136K,I178L,N180H,K229T,P256S,V292I,A586D,V621I,S683G	181
S31T,T41I,S42P,-44T,T93M,T107I,M110T,N136K,V144I,A204E,T233N,R235H,E238N,F335Y D542N,A586D	145
S42P,-44S,A62V,M110T,I178L,E257D,V292I,A586D	125
S42P,-44S,P56S,E80K,G82D,M110T,N114D,N136K,Q145L,P179T,P186T,T208S,T233N,R235H,V292I, K438Q, A586D,D626N,I641L,A662S,T669I,R677S,S683G	105
S42P,A48V,S96L,H99Y,M110T,I178L,A586D	66
S42P,-44S,M110T,Q127H,I178L,D210E,A586D	61
V29I,S42L,-44S,I108S,M110T,N136K,V292I,A586D,S683G	57
N81D,M110T	54
M110T,A586D	48
S42P,-44S,P56S,E80K,G82D,M110T,N114D,N136K,Q145L,P179T,P186T,T208S,T233N,R235H,A285-, V292I,K438Q,A586D,D626N,I641L,A662S,T669I,R677S,S683G	46
M110T,R522H,A586D	32
S42P,-44S,M110T,A133V,I178L,V292I,L295I,A586D	32
S42P,M110T,N136K,P191T,K343N,A586D	27
V29I,S42L,-44S,M110T,N114D,N136K,H148R,H249R,N254K,V292I,A586D,S683G	25
S42P,M110T,N136K,A222V,T233N,R235H,A286T,V292I,A586D	23
S42P,A48V,S96L,H99Y,M110T,I178L,K334N,A586D	21
S42P,-44S,M110T,I178L,E575A,A586D	18
V29I,S42P,-44S,G71R,M110T,N136K,A150T,I178L,A586D	17
S42P,-44S,M110T,T158R,S171F,I178L,I226T,V292I,A586D	15
S42P,-44S,M110T,I178L,N214S,A586D,V621I	14
S42P,-44S,P56S,E80K,G82D,M110T,N114D,N136K,Q145L,P179T,P186T,T208S,T233N,R235H,V292I, K438Q,P446S,A586D,D626N,I641L,A662S,T669I,R677S,S683G	13
M110T,E575G	12
S42P,-44S,H99Q,M110T,H129N,G154R,I178L,P186T,V292I,A586D	10
S42P,-44S,M110T,A133V,I178L,A198E,V292I,L295I,R362C,A586D	8
M110T,A207E	6
S42P,-44S,M110T,S128P,I178L,A586D,V621I	6
S42P,-44S,M110T,I178L,A297E,E575A,A586D	5
S42P,-44S,M110T,A133V,I178L,H223Y,V292I,L295I,A586D	4
V29I,S42P,-44S,T86I,M110T,N114D,Q130P,N136K,I172V,T208S,T233N,R235H,V292I,K438Q,A586D, D626N,I641L,S683G	4
M110T,D272-,A273-,L274-,Q275-	4
S42P,-44S,N104I,M110T,I178L,A586D,V621I	4
S42P,-44S,M110T,A133V,I178L,V292I,L295I,A379V,A586D	4
V20L,V29I,S42P,-44S,A48T,M110T,A150T,S188P,P191T,K278N,A586D	4
S42P,A48V,S96L,H99Y,M110T,I178L,A204V,A586D	4
S42P,-44S,M110T,I178L,N214S,A324S,A586D,V621I	3
M110T,A174T	3
R8-,K9-,Y10-,S11-,I12-,S42P,-44S,M110T,I178L,E575A,A586D	3

Supplementary Table 2. MS/MS experiment of SWATH windows with m/z range, accumulation time (Acc. time) and collision energy (CE).

			ESI (+)			ESI (-)		
**Experiment**	**Scan type**	**Acc. Time (ms)**	**Start (m/z)**	**Stop (m/z)**	**CE (V)**	**Start (m/z)**	**Stop (m/z)**	**CE (V)**
1	MS Full Scan	50	50.0	1250.0	10	50	1250.0	-10
2	SWATH	31	50.0	217.6	45±15	50	213.5	-45±15
3	SWATH	31	216.6	340.3	45±15	212.5	271.4	-45±15
4	SWATH	31	339.3	441.4	45±15	270.4	314.6	-45±15
5	SWATH	31	440.4	524.9	45±15	313.6	382.6	-45±15
6	SWATH	31	523.9	571.6	45±15	381.6	427.5	-45±15
7	SWATH	31	570.6	643.4	45±15	426.5	464.3	-45±15
8	SWATH	31	642.4	687.3	45±15	463.3	501.0	-45±15
9	SWATH	31	686.3	720.1	45±15	500.0	540.8	-45±15
10	SWATH	31	719.1	740.1	45±15	539.8	617.5	-45±15
11	SWATH	31	739.1	755.0	45±15	616.5	680.3	-45±15
12	SWATH	31	754.0	764.1	45±15	679.3	697.1	-45±15
13	SWATH	31	763.1	775.1	45±15	696.1	724.0	-45±15
14	SWATH	31	774.1	786.1	45±15	723.0	749.0	-45±15
15	SWATH	31	785.1	793.1	45±15	748.0	775.6	-45±15
16	SWATH	31	792.1	806.1	45±15	774.6	793.1	-45±15
17	SWATH	31	805.1	814.2	45±15	792.1	811.0	-45±15
18	SWATH	31	813.2	829.6	45±15	810.0	832.6	-45±15
19	SWATH	31	828.6	842.7	45±15	831.6	854.1	-45±15
20	SWATH	31	841.7	903.3	45±15	853.1	861.2	-45±15
21	SWATH	31	902.3	1250.0	45±15	860.2	1050.0	-45±15
