# Supplementary material for: Lipase-mediated detoxification of host-derived antimicrobial fatty acids by Staphylococcus aureus
Source: Commun Biol. 2024 May 15;7:572. doi: 10.1038/s42003-024-06278-3 (PMC11096360; doi:10.1038/s42003-024-06278-3)
Supplement: Supplementary file 3 — Description of additional supplementary files [file 42003_2024_6278_MOESM3_ESM.docx]

Description of Additional Supplementary Files

**File name:** Supplementary Data 1

**Description:** The source data for graphs and charts in the paper
