# Supplementary material for: Lipase-mediated detoxification of host-derived antimicrobial fatty acids by Staphylococcus aureus
Source: Commun Biol. 2024 May 15;7:572. doi: 10.1038/s42003-024-06278-3 (PMC11096360; doi:10.1038/s42003-024-06278-3)
Supplement: Supplementary file 5 — Reporting summary [file 42003_2024_6278_MOESM5_ESM.pdf]

nature portfolio

Corresponding author(s): Arnaud Kengmo Tchoupa

Last updated by author(s): Apr 18, 2024

Reporting Summary

Nature Portfolio wishes to improve the reproducibility of the work that we publish. This form provides structure for consistency and transparency in reporting. For further information on Nature Portfolio policies, see our Editorial Policies and the Editorial Policy Checklist.

Statistics

For all statistical analyses, confirm that the following items are present in the figure legend, table legend, main text, or Methods section.

n/a	Confirmed	
☐	☒	The exact sample size (n) for each experimental group/condition, given as a discrete number and unit of measurement
☐	☒	A statement on whether measurements were taken from distinct samples or whether the same sample was measured repeatedly
☐	☒	The statistical test(s) used AND whether they are one- or two-sided<br>*Only common tests should be described solely by name; describe more complex techniques in the Methods section.*
☒	☐	A description of all covariates tested
☐	☒	A description of any assumptions or corrections, such as tests of normality and adjustment for multiple comparisons
☐	☒	A full description of the statistical parameters including central tendency (e.g. means) or other basic estimates (e.g. regression coefficient) AND variation (e.g. standard deviation) or associated estimates of uncertainty (e.g. confidence intervals)
☐	☒	For null hypothesis testing, the test statistic (e.g. F, t, r) with confidence intervals, effect sizes, degrees of freedom and P value noted<br>*Give P values as exact values whenever suitable.*
☒	☐	For Bayesian analysis, information on the choice of priors and Markov chain Monte Carlo settings
☒	☐	For hierarchical and complex designs, identification of the appropriate level for tests and full reporting of outcomes
☒	☐	Estimates of effect sizes (e.g. Cohen's d, Pearson's r), indicating how they were calculated

Our web collection on statistics for biologists contains articles on many of the points above.

Software and code

Policy information about availability of computer code

Data collection	Optical density readings over 24 hours were recorded by the Gen5 software (Agilent BioTek)
Data analysis	Prism 9.5.1 (GraphPad) was used to analyze all data except for genomics. In-silico PCR to extact lip2 sequences was performed with a Perl script (https://github.com/egonozer/in_silico_pcr). PHASTER (https://phaster.ca/) was used to identify prophage sequences within lip2.

For manuscripts utilizing custom algorithms or software that are central to the research but not yet described in published literature, software must be made available to editors and reviewers. We strongly encourage code deposition in a community repository (e.g. GitHub). See the Nature Portfolio guidelines for submitting code & software for further information.

Data

Policy information about availability of data

All manuscripts must include a data availability statement. This statement should provide the following information, where applicable:

- Accession codes, unique identifiers, or web links for publicly available datasets
- A description of any restrictions on data availability
- For clinical datasets or third party data, please ensure that the statement adheres to our policy

Amplicons of the in-silico PCR of lip2 genes (https://doi.org/10.6084/m9.figshare.23828196.v1), multiple sequence alignment of Lip2 (https://doi.org/10.6084/m9.figshare.23828274.v1) and prophage sequences within lip2 (https://doi.org/10.6084/m9.figshare.23828274.v1) are available on Figshare. All the other data are available within the article and its supplementary materials.

Research involving human participants, their data, or biological material

Policy information about studies with human participants or human data. See also policy information about sex, gender (identity/presentation), and sexual orientation and race, ethnicity and racism.

Reporting on sex and gender	NA
Reporting on race, ethnicity, or other socially relevant groupings	NA
Population characteristics	NA
Recruitment	NA
Ethics oversight	NA

Note that full information on the approval of the study protocol must also be provided in the manuscript.

Field-specific reporting

Please select the one below that is the best fit for your research. If you are not sure, read the appropriate sections before making your selection.

☒ Life sciences ☐ Behavioural & social sciences ☐ Ecological, evolutionary & environmental sciences

For a reference copy of the document with all sections, see nature.com/documents/nr-reporting-summary-flat.pdf

Life sciences study design

All studies must disclose on these points even when the disclosure is negative.

Sample size	For in vivo experiments, five mice were used per group. Two discs soaked with bacteria were applied to back skin of each mouse for a total of n = 10 per group. This is commonly used in the field and enabled robust statistical analysis.
Data exclusions	One sample was excluded because the mouse managed to dislodge the disc with bacteria.
Replication	Each experiment was either repeated at least three times or included samples generated on at least three independent occasions.
Randomization	Mice were randomly assigned to experimental groups.
Blinding	Blinding was not relevant to our study.

Reporting for specific materials, systems and methods

We require information from authors about some types of materials, experimental systems and methods used in many studies. Here, indicate whether each material, system or method listed is relevant to your study. If you are not sure if a list item applies to your research, read the appropriate section before selecting a response.

Materials & experimental systems

n/a	Involved in the study	
☒	☐	Antibodies
☒	☐	Eukaryotic cell lines
☒	☐	Palaeontology and archaeology
☐	☒	Animals and other organisms
☒	☐	Clinical data
☒	☐	Dual use research of concern
☒	☐	Plants

Methods

n/a	Involved in the study	
☒	☐	ChIP-seq
☐	☒	Flow cytometry
☒	☐	MRI-based neuroimaging

Animals and other research organisms

Policy information about studies involving animals; ARRIVE guidelines recommended for reporting animal research, and Sex and Gender in Research

Laboratory animals	8 week-old female C57BL/6J mice were used.
Wild animals	NA
Reporting on sex	Only female mice were used.
Field-collected samples	NA
Ethics oversight	Animal Ethics Committees of the Regierungspräsidium Tübingen (IMIT 3/18G)

Note that full information on the approval of the study protocol must also be provided in the manuscript.

Plants

Seed stocks	NA
Novel plant genotypes	NA
Authentication	NA

Flow Cytometry

Plots

Confirm that:

☐ The axis labels state the marker and fluorochrome used (e.g. CD4-FITC).

☐ The axis scales are clearly visible. Include numbers along axes only for bottom left plot of group (a 'group' is an analysis of identical markers).

☐ All plots are contour plots with outliers or pseudocolor plots.

☐ A numerical value for number of cells or percentage (with statistics) is provided.

Methodology

Sample preparation	Bacteria were stained with azide fluor 488 alone or with palmitoleic alkyne (click chemistry). After extensive wash with PBS, the fluorescence of 10,000 bacteria per sample was measured.
Instrument	BD LSRFortessa flow cytometer
Software	BD FACS DIVA Version 8; FlowJo v10.8.1
Cell population abundance	Samples were not divided into subpopulations
Gating strategy	No gating strategy way used.

☐ Tick this box to confirm that a figure exemplifying the gating strategy is provided in the Supplementary Information.
